# Is autologous platelet-rich plasma capable of increasing hair density in patients with androgenic alopecia? A systematic review and meta-analysis of randomized clinical trials^⋆^

**DOI:** 10.1016/j.abd.2024.01.002

**Published:** 2024-07-15

**Authors:** Lucas Kieling, Ana Terezinha Konzen, Rafaela Koehler Zanella, Denis Souto Valente

**Affiliations:** a Division of Surgical Clinics, Universidade Federal de Ciências da Saúde de Porto Alegre, Porto Alegre, RS, Brazil; b Graduate Program in Medicine and Health Sciences, Faculdade de Medicina da Pontifícia, Universidade Católica do Rio Grande do Sul, Porto Alegre, Brazil

**Keywords:** Alopecia, Meta-analysis, Platelet-rich plasma, Systematic review

## Abstract

**Fundamentals:**

Platelet-rich plasma (PRP) has been progressively more used in androgenetic alopecia (AGA).

**Objectives:**

The authors aimed to evaluate PRP efficacy compared to placebo in AGA.

**Methods:**

A comprehensive search was conducted across seven databases, until 01/04/2023. Randomized clinical trials focusing on AGA and PRP use to increase hair density were included. Patients aged between 15 and 63 years, diagnosed with AGA characterized by Norwood I‒VII and Ludwig I‒III scales, were included. Studies with a sample size <10, lacking PRP processing method, focusing on complementary therapies or other alopecias, were excluded. The authors conducted subgroup analysis for activator, spin method, study design, risk of bias, and gender. Meta-regression was conducted for activator, spin method, design, and gender. The authors used GRADEpro to assess evidence certainty and the RoB-2 tool for risk of bias. Asymmetry was measured through a Funnel plot followed by Egger’s test. The protocol was registered at PROSPERO (CRD42023407334).

**Results:**

The authors screened 555 registers and included fourteen studies involving 431 patients for qualitative synthesis, with 13 studies included in the meta-analysis. Meta-analysis demonstrated a mean difference of 27.55 hairs/cm^2^ and 95% CI (14.04; 41.06), I^2^ = 95.99%, p < 0.05. Hair diameter meta-analysis presented a mean difference of 2.02 μm, 95% CI (−0.85 μm; 4.88 μm), and I^2^ = 77.11% (p = 0.02). That is, low quality evidence.

**Study limitations:**

Studies were highly heterogeneous, of low quality, and presented evident publication bias.

**Conclusions:**

Highly heterogeneous studies with publication bias suggest PRP effectively increases hair density in AGA, so further high-quality randomized clinical trials are recommended to strengthen the evidence.

## Introduction

Androgenetic Alopecia (AGA) is a common condition characterized by progressive hair loss and decreased hair density, impacting hair color, diameter, and length. It is a polygenic condition that affects a significant proportion of the population, with up to 80% of men and 50% of women experiencing it at some point in life.^1^, ^2^

In men, the pattern of hair loss typically involves recession in the temporal regions and thinning at the vertex, resulting in a characteristic horseshoe-shaped hair pattern.^3^, ^4^ In contrast, women tend to experience hair loss in the mid-frontal area, with a delayed onset and less overall density loss.^5^, ^6^

Hair significance and its impact on individuals' lives shouldn’t be underestimated. Hair is one of the first characteristics the authors notice in others, and studies have shown that hair loss can impact an individual's social life and overall lifestyle.^7^, ^8^ The condition of our hair plays a crucial role in our self-perception and attractiveness, influencing our social interactions and opportunities.^9^ Therefore, understanding hair loss and its treatment options is essential in improving quality of life.

Platelet-Rich Plasma (PRP) is a concentrated form of plasma obtained from a patient's own blood, containing higher levels of platelets compared to baseline levels.^10^ Various techniques for preparing PRP exist, although a consensus has yet to be reached.^11^ PRP has been utilized for many years in regenerative medicine, particularly in osteoarthritic injury treatment and rotator cuff damage. Recently, its use has expanded, PRP is now world widely performed by dermatologists for several indications, such as skin rejuvenation, and AGA treatment.^12^, ^13^, ^14^, ^15^

PRP action on the scalp is related to the growth factors present in the concentrated platelets.^16^ For example, the Vascular Endothelial Growth Factor (VEGF) stimulates the dermal papilla cells through the VEGFR-2/ERK pathway.^17^ Insulin-like Growth Factor 1 (IGF-1) and Fibroblast Growth Factor (FGF) promote the anagen phase of hair follicles and inhibit the transition to the catagen phase.^18^, ^19^, ^20^ Additionally, Epidermal Growth Factor (EGF) induces the proliferation of dermal papilla cells via the Notch signaling pathway and has been shown to promote hair growth in experimental studies.^21^, ^22^ Platelet-Derived Growth Factor (PDGF) also induces and maintains hair follicles in the anagen phase in animal models.^23^, ^24^

Systematic reviews suggest that PRP increases mean hair density in male and female pattern hair loss and on alopecia areata.^25^, ^26^ Although some were exclusively on clinical trials, evidence quality is persistently low.^27^, ^28^, ^29^ Non-randomized clinical trials are indeed inferior to randomized clinical trials. As such, they are considered high-tiered evidence quality suppliers, and, if assessed separately, may provide better insights on existent evidence quality.

Despite the increase in research and use of PRP for androgenetic alopecia, there is currently no consensus based on substantial evidence from randomized clinical trials. Accordingly, this study’s objective is to evaluate the existing evidence regarding the application of PRP injections to increase hair density in patients with AGA, specifically through a review of randomized clinical trials.

## Methods

### Study design

This study employed a systematic review and meta-analysis methodology, adhering to the guidelines outlined in the PRISMA (Preferred Reporting Items for Systematic Reviews and Meta-Analyses) checklist.

### Eligibility criteria

The research question was created using the PICO format: among patients with androgenetic alopecia, does autologous platelet-rich plasma, compared to a placebo, result in hair density increase in randomized clinical trials?

To be included in the analysis, studies needed to meet the following eligibility criteria. Firstly, only full-length original articles reporting on randomized clinical trials were considered. The intervention of interest was the administration of Platelet-Rich Plasma (PRP) injections. PRP can either be activated or non-activated. The use of PRP in combination with the platelet activator/agonist allows for the release of multiple growth factors and differentiation factors upon platelet activation, which can enhance the process.^30^ Activation can be done by adding a platelet activator/agonist as topical bovine thrombin or 10% calcium chloride to the PRP solution right before injection. The control group consisted of individuals who received a placebo. This is to control PRP efficacy. Comparison with other standard therapies, such as minoxidil or 5-alpha-reductase inhibitors, could contaminate data and provide non-representative results. If PRP doesn’t show benefit compared to placebo, there is no point in comparing it to other therapies. Our main outcome of interest was changes, either positive or negative, in hair density. It refers to changes in the number of hairs per unit area (cm^2^) on the skin surface. Changes in hair diameter were secondary outcomes.

The target population comprised patients aged between 15 and 63 years diagnosed with AGA, as is the most likely population to be affected by androgenetic alopecia, who would potentially search for and benefit from PRP interventions. Secondly, women and men can be included, as androgenetic alopecia can affect both genders. While it’s more prevalent in men, women can also experience this condition. Characterization by the Norwood I‒VII and Ludwig I‒III classifications. Some studies use inter-patient and others use intra-patient comparison; in this review, both could be included. Studies with a sample size of fewer than ten patients, those lacking information on the PRP processing method, or studies that subjectively assessed increased hair density were excluded.

Furthermore, patients with chronic diseases, individuals on anticoagulant therapy, pregnant women, those with malignancies, and those utilizing complementary therapies were also excluded. Additionally, studies focusing on other types of alopecia, such as alopecia areata, cicatricial alopecia, or lichen planus, were not considered. There were no limitations regarding the PRP processing method.

### Data sources

The authors conducted a comprehensive search until April 1, 2023, in seven databases. These databases included MEDLINE (through PubMed), Virtual Health Library (BVS), EMBASE, Cochrane Library, Web of Science, Scopus, and MedRxiv (a source of grey literature, encompassing pre-print studies). The selection of these databases aimed to capture a wide range of relevant studies for inclusion in the review and meta-analysis. References from studies cited in this review writing process were also screened and eligible for inclusion.

### Search strategy

For the search in electronic databases, relevant Medical Subject Headings (MeSH) terms and keywords were utilized. These terms were adapted based on the specific database. The following terms were employed to ensure a comprehensive search: “Hair regrowth”, “Hair restoration”, “Hair regeneration”, “Hair Growth”, “Alopecia”, “Baldness”, “Hair Loss”, “Hair Losses”, “Loss, Hair”, “Losses, Hair”, “Alopecia, Male Pattern”, “Male Pattern Alopecia”, “Baldness, Male Pattern”, “Male Pattern Baldness”, “Female Pattern Baldness”, “Baldness, Female Pattern”, “Androgenetic Alopecia”, “Pattern Baldness”, “Baldness”, “Pattern”, “Androgenic Alopecia”, “Alopecia, Androgenic”, “Alopecias, Androgenic”, “Pseudopelade”, “Alopecia Cicatrisata”, “Alopecia Cicatrisatas”, “Platelet-Rich Plasma”, “Plasma, Platelet-Rich”, “PRP”, “thrombocyte rich plasma”, and “Randomized Clinical Trial (Publication Type)”. To improve the sensitivity for identifying randomized clinical trials, recommendations from McGill University were followed.^31^ Specific search strategies for each database utilized in this study can be found in the supplementary file.

### Study selection

Two independent reviewers conducted the assessment of titles and abstracts for all identified clinical trial reports. Blinding was ensured, with each reviewer unaware of the decisions made by the other. When possible, studies that met the inclusion criteria were selected for further evaluation. In cases of disagreement between reviewers, a third reviewer was involved to make the final decision regarding study inclusion or exclusion. The Rayyan software was utilized to facilitate the selection process, particularly for reading and screening titles and abstracts.

### Data extraction

Each reviewer independently performed data extraction without access to the other reviewer's extraction. The following data were extracted from the selected studies: author, year, location, comparator, study design, sample size, follow-up time, method of PRP preparation, capillary density and diameter measurements, and funding sources. A predefined Excel spreadsheet was used to record the extracted data for each reviewer.

### Assessment of methodological quality of selected articles for systematic review

Risk of bias assessment was conducted using the RoB-2 tool provided by Cochrane. This tool assesses possible biases in five domains, including randomization, intended intervention, missing data, outcome measurement, and outcome reporting.^32^ The GRADE method was employed to assess the strength of the recommendation for the intervention.^33^

### Data analysis

Included studies were grouped based on the characteristics provided in Table 1. The extracted data were qualitatively synthesized and recorded in a dedicated spreadsheet. For studies that provided the mean and standard deviation of the outcomes of interest, a random-effects meta-analysis was conducted using the restricted maximum likelihood method. When these data were absent, authors were contacted. When authors didn’t answer, if provided sufficient data for estimating the mean and standard deviation, the study would be included in the meta-analysis. The effect size and 95% Confidence Intervals (95% CI) were measured by the mean difference. Statistical heterogeneity was assessed using the I² statistic, with values below 25% considered low, between 25% and 50% considered moderate, and above 50% considered high. Subgroup analysis and meta-regression were performed based on the use of a pre-application activator, sex, study design, risk of bias and spin method following the aforementioned meta-analytic model. Statistical package STATA® version 16 was used for data analysis. Funnel plots followed by Egger’s test were done to assess asymmetry and publication bias.

**Table 1:** Study characteristics.

Characteristics	Sample (n)	%
**Design**		
Inter-subject comparison group	8	57.14%
Intra-subject comparison group	6	42.86%
**Use of activator**		
-Active PRP	7	50.00%
-Non-active PRP	7	50.00%
**Geographical location**		
America	5	35.71%
Asia	4	28.57%
Africa	2	14.29%
Oceania	0	
Europa	3	21.43%
**Increased Hair density**		
Yes	13	92.86%
No	1	7.14%
**Increased Hair diameter**		
Yes	5	35.71%
No	2	14.29%
Not measured	7	50.00%
**Publication Year**		
Before 2019	5	35.71%
2019-2023	9	64.29%
**High Grade alopecia (Stage > IV NH and > II Ludwig)**		
Included	9	64.29%
Excluded	5	35.71%

### Data estimation

Gressenberger P:^34^ The Interquartile Range (IQR) was calculated from the reported range as Range/4. This IQR value was then used to estimate the standard deviation according to the empirical rule relationship of IQR/1.349. The median was used as an initial point estimate for the mean. From these estimates, the variance was determined as the square of the standard deviation. Finally, a 95% CI was placed around the estimated mean using a *t*-distribution, with degrees of freedom based on the sample size. This process allows approximate quantification of the mean and variation based on limited summary statistics when the full dataset is not available for direct calculation.

Chuah SY:^35^ Hair density values at 6 months were calculated by taking the baseline values and adding or subtracting reported difference values. Standard deviations were also adjusted to account for variability in the different groups. Specifically, the baseline means, and standard deviation were used as a starting point. Then, the difference in means was either added or subtracted based on whether the difference was positive or negative. The difference standard deviation was also added or subtracted to the baseline standard deviation. This allowed the full range of values to be determined while incorporating the variability represented by the CI. Standard Deviation (SD) was calculated by: SD = √(Baseline SD + Difference SD).

Dubin DP:^36^ The baseline range was used to estimate the standard deviation for each group according to the formula of range/4 * (sample size/(sample size - 1)). The mean difference from the baseline was then added to the original baseline mean to calculate the estimated post-treatment mean. The baseline standard deviation was assumed to apply after treatment. Finally, a 95% CI was placed around each estimated post-treatment mean using the *t*-distribution, with degrees of freedom based on the sample size and estimated standard deviation. This process allowed the estimation of key metrics after the intervention based solely on summary baseline data and mean treatment differences reported for each experimental condition.

**Fig. 1:**
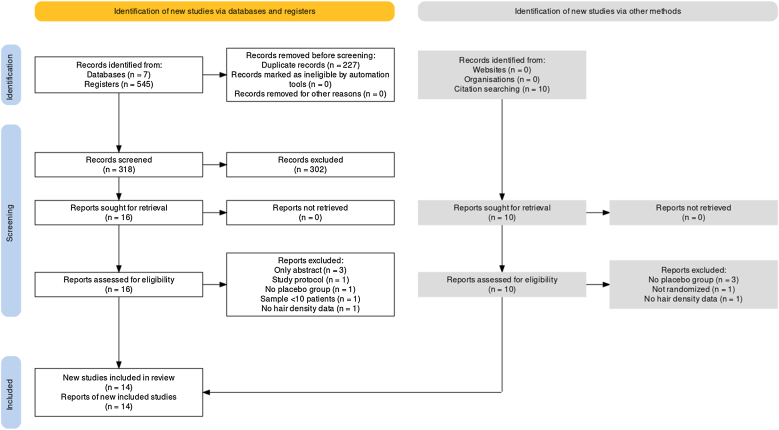
Prisma flow diagram.

Rodrigues BL:^37^ The authors extracted data from Fig. 1 with WebPlotDigitizer (Ankit Rohatgi, version 4.6, 2022). Then, the mean and standard deviation were calculated based on Luo et al. and Shi et al. works to estimate mean and standard deviation from the sample size, minimum value, first interquartile, median, third interquartile, and maximum.^38^, ^39^

The authors couldn’t estimate or retrieve data from Siah TW et al., therefore this report has only been included in qualitative synthesis, without participation in the meta-analysis model.^40^

### Research protocol

The research protocol was registered at PROSPERO (International Prospective Register of Systematic Reviews) under the registration number CRD42023407334.

## Results

### Selected studies

Following a comprehensive database search, 545 records were identified. Among them, the authors removed 227 duplicates, leaving 318 papers for full-text screening. After careful reference screening, 10 registers were also added. Of these, 26 papers were potentially eligible for inclusion. All reports were retrieved. Ultimately, 14 of them were included in the qualitative synthesis.^34^, ^35^, ^36^, ^37^, ^40^, ^41^, ^42^, ^43^, ^44^, ^45^, ^46^, ^47^, ^48^, ^49^ Among the included studies, 13 provided sufficient data for quantitative synthesis through meta-analysis. It’s important to note that some studies had mean and standard deviation estimated as explained in the methods section. Supplementary Material contains the excluded reports list. Fig. 1 presents the PRISMA flow diagram.

The review included 431 participants, with 319 subjects undergoing PRP intervention and 310 individuals receiving a placebo. Intra-subject participants were considered in both groups. Most randomized clinical trials were conducted after 2019 and utilized inter-subject comparisons. Half used pre-application activators. Studies included patients with all degrees of alopecia. Table 1 summarizes the included studies' characteristics.

The majority of the studies used the trichoscan device and patient photos to aid in the analysis and measurement of capillary density as an outcome. Regarding PRP application, most studies administered monthly injections at a rate of 0.1‒0.2 mL/cm^2^, although Tawfik AA et al. achieved better results with weekly injections.^41^ The method of obtaining and applying PPR is presented for each study in Table 2.

**Table 2:** Study Summary.

Author and Year	Financing	Study design	Comparator	Sample	Follow up time	PRP method	Results
Shapiro, 2020	Regen Labs SA (Le Mont-sur-Lausanne, Switzerland)	Randomized clinical trial, Half-Scalp, intra-subject	Placebo controlled (saline)	35, 17 female and 18 male	1 application per month for 3 months, results were measured a month after treatment ended. 0.1‒0.2 mL/cm^2^ of PRP used	10 mL of blood centrifuged at 1500 g, using a thixotropic gel to prepare approximately 5 mL of PRP	↑ Hair density (NSDBG); ↑ Hair diameter (NSDBG)
Gressenberger, 2020		Single-center, randomized, blinded pilot study inter-subject	Placebo-controlled (saline)	30 male. 20 PRP and 10 placebo	Five treatments were performed with 0.1 mL/cm^2^ of PRP, at intervals of 4–6 weeks. Hair density measured at baseline, 4 weeks, and 6 months after treatment	20 mL of blood collected in a tube containing sodium citrate. used a single spin procedure (2800 RPM for 9 minutes)	↓ Hair density (NSDBG); ↓ Hair diameter
Siah, 2020	Supported by unrestricted funds from RepliCel Life Sciences Inc, Vancouver, Canada	Randomized, intra-subject	Placebo-controlled (saline)	10, 9 female and 1 male	Five injections with 2 weeks interval each, 0.1 mL/cm^2^ of PRP was used. Measurement at baseline, after 4 weeks (prior to 3^rd^application) and 8 weeks after treatment end	20 mL of venous blood was centrifuged at 300 g at 18 °C for 5 minutes, PRP1 transferred and centrifuged at 700 g at 18 °C for 17 minutes, after that Platelet-Poor Plasma (PPP) was removed and resuspended to form PRP2	↑ Hair density (NSDBG)
Singh, 2020	Institutional Research Grant	Single-center, prospective, randomized, double-blind control trial, inter-subject	Minoxidil 5%, Placebo (saline), Minoxidil + PRP	80. all male. 20 Minoxidil 5% + PRP, 20 Minoxidil 5%, 20 PRP, and 20 saline	0.05–0.1 mL/cm^2^ of prp was injected, treatment done 3 times with 1 month interval between each. Measurement before treatment and documented every month for 5 months	18 mL of blood collected into a 2 mL tube with 2.8% of sodium citrate. Centrifugation at 2200 rpm for 12 minutes, separation of buffy coat and plasma to 2^nd^spin at 3000 rpm for 6 minutes. 3–3.5 mL of PRP was obtained. PRP was added to a syringe with calcium gluconate (1:9) as an activator	↑ Hair density (NSDBG)
Dicle, 2018	Akdeniz University, Scientific Research Projects Coordination Unit, Antalya, Turkey	Randomized, placebo‐controlled, inter-subject crossover study	Placebo-controlled (saline)	25 male G1 = 10, G2 = 15	Monthly application for 3 months, after that, there was a 3-month wash-out. Then, crossover happened and placebo group received PRP. Patients were evaluated three times: at baseline (M0), at 4^th^month (M4), and 9^th^month (M9)	A 28-cc blood sample was collected and mixed with citrate dextrose-A (ACD-A). 30 cc was transferred to a PRP tube and centrifuged at 1700 G for 5 minutes. 5 mL of PRP was loaded into a syringe and activated using a bio-activator before application	↑ Hair density (SDBG)
Rodrigues, 2019	Supported by the National Council of Technological and Scientific Development	Double-blinded investigative pilot prospective study, randomized, inter-subject	Placebo controlled (saline)	26 male. PRP group (n = 15) and the control group (n = 11)	4 applications every 15 days, with a total of 20 injections. 100 u L of PRP or saline solution per injection, total of 2 mL. Hair density measured before the treatment, 15 days and 3 months after end of treatment	Blood was collected in six 8.5-mL acid citrate dextrose tubes, one 4-mL ethylenediaminetetraacetic acid tube, and 1 tube without anticoagulant. Centrifuged at 300 g for 5 minutes at 18 °C, and the upper fraction (PRP1) was separated without disturbing the buffy coat. PRP1 was centrifuged at 700×g for 17 minutes at 18 °C. The platelet pellet from PRP1 was resuspended in 300 μL PPP (PRP2). 20 mM of CaCl2 and 25 IU/mL human thrombin or CaCl2 alone were used to activate. Incubation at 37 °C for 1 hour and at 4 °C for 16 hours. The activated PRP2 was recovered by centrifugation at 3,000×g for 20 minutes at 18 °C. Autologous serum was used as an activator and prepared from the tube without anticoagulant: after centrifugation at 1258 g for 15 minutes, 1 mL was separated and then added to the PRP	↑ Hair density (Group 1 NSDBG; Group 2 SDBG)
Gentile, 2015		Randomized, evaluator-blinded, intra-subject	Placebo-controlled (saline)	23 male	0.1 mL/cm^2^ of PRP was applied monthly for 3 months (3 injections). Evaluation happened before treatment (T0), 2(T1), 6(T2), 12(T3), 16(T4) and 24 (T5) months	18 mL of blood were taken and centrifuged in two tubes containing sodium citrate at 1,100 g for 10 minutes. Then, autologous PRP that was not activated after centrifugation (9 mL) was switched to 10-mL tubes containing Ca2+ extracted by the Cascade-Selphyl-Esforax	↑ Hair density (SDBG);
Tawfik, 2018		Randomized, double-blinded, intra-subject	Placebo-controlled (saline)	30 female	4 treatments were done on each patient, every week. Patients were followed up at 6 months after the last session	10 mL blood was collected, with 1.5 mL of sodium citrate as an anticoagulant. Then, centrifuged at 1200 g for 15 minutes. Buffy coat was separated together with platelet poor plasma and centrifuged at 2000 g for 10 minutes. PPP was discarded and platelet concentrate was loaded into a 1-mL insulin syringes containing calcium gluconate in a 1:9 ratio (0.1 mL calcium gluconate per 0.9 mL of PRP)	↑ Hair density (SDBG); ↑ Hair diameter (SDBG);
Alves, 2016		Randomized, double-blinded, intra-subject	Placebo-controlled (saline)	25, 12 men and 13 women. 22 of those completed the treatment (11:11)	Monthly application of 0.15 mL/cm^2^ for 3 months of patients were evaluated before, after second, third treatment and after 6 months of treatment end	18 mL of blood was transferred to a tube with 2 mL of 3.8% sodium citrate. Then, centrifuged at 460 g for 8 minutes. PPP was discarded and 3 mL of PRP was obtained. PRP was activated with 0.15 mL of 10% calcium chloride	↑ Hair density (SDBG)
Qu, 2021	National Natural Science Foundation of China	Randomized, double-blinded, intra-subject	Placebo-controlled (saline)	32 Male; 20 female	PRP was injected on half-head of the alopecia areas in 0.05- to 0.1-mL; Monthly session for 3 m; follow up 0, 3 m and 6 m	40 mL of blood in the Tricell kit. 310 g for 4 min and next 300 g for 3 min	↑ Hair density (SDBG); ↑ Hair diameter (SDBG);
Chuah, 2021		randomized, double-blind, intra-subject	Placebo-controlled (saline)	32 male, 18 female	4 sessions, three weeks interval. evaluation at 0, 3 m, and 6 m	centrifugated 5 min at 1500 g, tube contained sodium citrate solution, as anticoagulant, Removal of PPP. Activation with calcium gluconate 1:9	↑ Hair density (NSDBG); ↑ Hair diameter (SDBG);
Toama, 2017		Randomized, inter-patient	Placebo-controlled (saline)	40 (PRP (11 males and 9 females) SALINE (8 males and 12 females)	5 sessions (one session every two weeks). Measured at 0; 3 m, and 6 m	Tubes with anticoagulant Na Citrate 3.8%. Centrifuged at 1,700 rpm for 15 minutes; centrifuged again at 3,000 rpm for 10 minutes; discard PPP; activation by calcium chloride 1:9	↑ Hair density (SDBG);
Dubin, 2020	Eclipse Aesthetics, LLC	Randomized, double-blind, inter-patient	Placebo-controlled (saline)	30 female (15 PRP)	6 m evaluation; 3 monthly injections	3,500 revolutions per minute for 10 minutes. PPP removed. The remaining 4.0 mL of supernatant was gently inverted in the vacutainer 7 times to re-suspend the platelets within the plasma	↑ Hair density (SDBG); ↑ Hair diameter (SDBG);
Gentile, 2017		Randomized, double-blind, intra-patient	Placebo-controlled (saline)	18 male (9 PRP)	3 m evaluation; 3 monthly injections	Centrifugation (1200 rpm for 10 min)	↑ Hair density (SDBG);

NSDBG, Not Significant Difference Between Groups; SDBG, Significant Difference Between Groups.

**Fig. 2:**
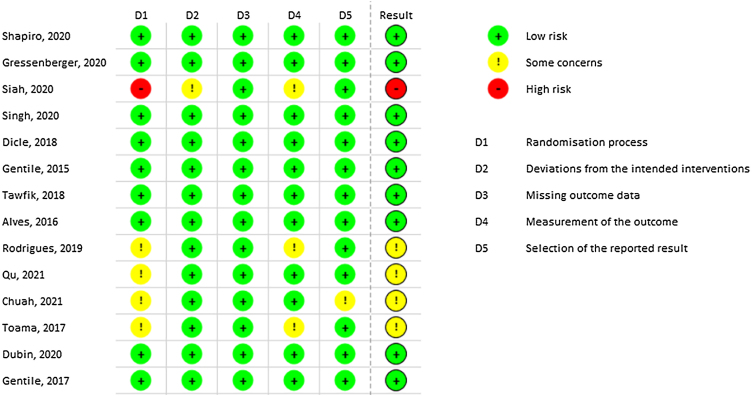
Rob-2 overall risk of bias.

Most of the included studies demonstrated a low risk of bias. However, Siah TW et al. exhibited a high risk of bias due to the lack of explanation regarding the randomization process.^40^ Similarly, Rodrigues BL et al. presented some risks in domains 1 and 4 related to randomization and outcome measurement, respectively.^37^ Qu Q, Chuah SY, and Toama MA also presented some concerns related to randomization, as no information from concealment was provided.^35^, ^48^, ^49^ Fig. 2 demonstrates all results from RoB-2 risk of bias.

### Hair density

Among the fourteen studies included in the synthesis, thirteen demonstrated improved hair density compared to the pretreatment baseline. However, Gressenberger P et al. reported worsening hair conditions without a significant difference between the groups.^34^ Around 57% of the studies showed a statistically significant difference in hair density between treated and placebo groups.

**Fig. 3:**
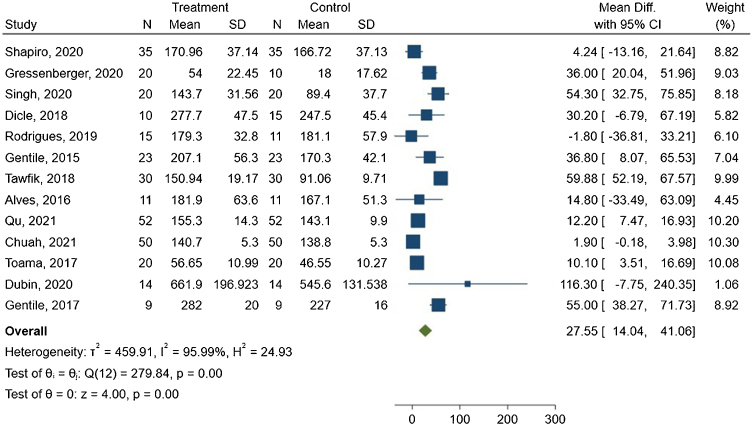
Hair density forest plot.

The meta-analysis showed a mean difference of 27.55 hairs/cm^2^ between the groups, with 95% CI ranging from 14.04–41.06. The forest plot is presented in Fig. 3.

The I^2^ statistic indicated high heterogeneity among the studies, with a value of 95.99% and Q(12) = 279.84. This result was statistically significant (p < 0.00).

In subgroup analysis, reports that used active PRP demonstrated a slightly lower mean difference of 25.25 hairs/cm^2^, with 95% CI ranging from 4.89 to 45.61, I² of 96.78%, p < 0.05, and Q(6) = 225.89. At the same time, studies using inactive PRP showed a similar mean difference of 29.79 hairs/cm^2^, with a 95% CI ranging from 11.16 to 48.41, I^2^ of 85.98%, p < 0.05, and Q(5) = 35.55.

Subgroup analysis of PRP preparation related to the spin method was also conducted. Both single and double-spin preparations were effective in increasing hair density (p < 0.05). Subgroup analysis was also performed considering study design, inter or intra-subject. Inter-subject comparisons presented a mean difference of 33.72 hairs/cm^2^, I^2^ = 78.19, 95% CI from 14.04 to 49.68 (p < 0.05), and Q(7) = 44.20. Intra-subject presented worse results, with a mean difference of 19.20 hairs/cm^2^, I^2^ = 98.46%, 95% CI from −4.02 to 42.43 (p = 0.10), and Q(4) = 209.61. The last subgroup was gender-related studies. Only two studies with only females presented sufficient data for meta-analysis, which limits data pooling. Male-only studies had a mean difference of 39.12, I² = 53.65%, 95% CI from 24.94 to 53.30 (p = 0.06), and Q(5) = 10.64. Mixed studies had a mean difference of 7.39, I^2^ = 73.01%, 95% CI from 1.57 to 13.22 (p < 0.05), and Q(4) = 18.96. High risk of bias presented mean hair density increase = 7.34 n/cm^2^, I^2^ = 79.77%, 95% CI from 0.96 to 13.73, and Q(3) = 18.87. Low risk of bias mean increase was 39.98, I^2^ = 77.49%, 95% CI from 25.09 to 54.87, and Q(8) = 41.08. Both p < 0.00. Subgroup-exclusive Forest plots are available in the Supplementary Material.

### Hair diameter

**Fig. 4:**
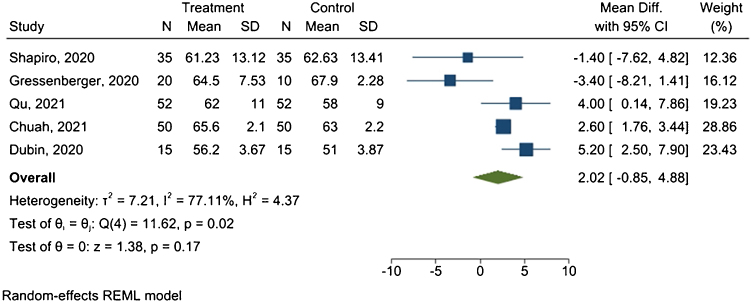
Hair diameter meta-analysis.

Five studies provided sufficient data for the meta-analysis. Fig. 4 presents the forest plot.

Results revealed a mean difference of 2.02 μm between treated and placebo groups, with 95% CI ranging from −0.85 μm to 4.88 μm, and I^2^ = 77.11% (p = 0.02).

### Asymmetry and publication bias

**Fig. 5:**
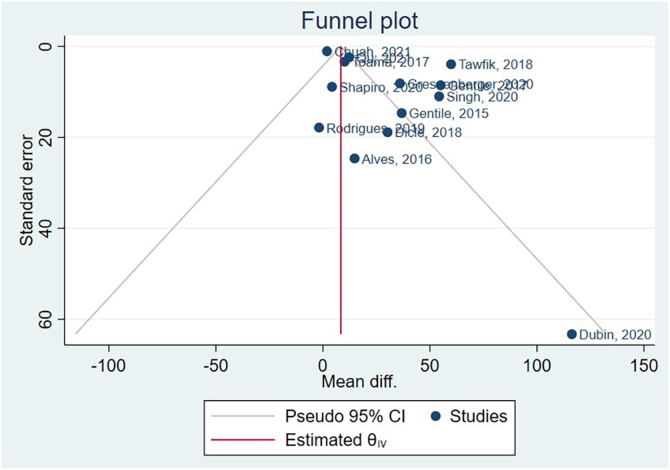
Funnel plot.

Hair diameter couldn’t have publication bias assessed, as there were only 5 studies, and the Cochrane Handbook suggests a minimum of 10 studies.^50^ The funnel plot generated after hair density meta-analysis is available in Fig. 5. Following, the authors conducted Egger’s test, beta1 = 0.83, SE of beta1 = 0.816, *z* = 1.01, Prob >|*z*| = 0.3115.

Based on these results, the coefficient beta1 is not statistically significant at the 5% level since the p-value is greater than 0.05. The standard error of beta1 is also quite high relative to the coefficient value. Although Egger’s test didn’t identify publication bias, funnel plot asymmetry results indicate a clear presence of publication bias. Supplementary Material presents specific subgroups funnel plots.

### GradePro assessment

The GRADEPro GDT analysis rated the evidence for PRP on hair density as low-quality evidence (++). This rating was based on a low overall risk of bias, high heterogeneity, direct outcomes, publication bias, and high precision in the results. As for hair diameter, it was assigned a very low-quality evidence rating (++). This rating was based on several factors, including a severe overall risk of bias, high heterogeneity, serious inconsistency, lack of direct outcomes, and imprecision in the results. Assessment can be seen in Table 3.

**Table 3:** GradePro assessment.

On androgenetic alopecia, does autologous platelet-rich plasma, compared to placebo, increases hair density and hair thickness, in randomized clinical trials?					
**Patient or population:** Androgenetic alopecia **Setting:** **Intervention:** Platelet-rich Plasma **Comparison:** Placebo					

Outcomes	№ of participants (studies) Follow-up	Certainty of the evidence (GRADE)	Relative effect (95% CI)	Anticipated absolute effects	
				Risk with Placebo	Risk difference with Platelet-rich Plasma
Incresead Hair Density assessed with: n/cm^2^	609 (13 RCTs)	⨁⨁◯◯ Low^a^	–		MD **27.55 hairs/cm² higher** (14.04 higher to 41.06 higher)
Hair Thickness	294 (5 RCTs)	⨁◯◯◯ Very low^b^	–		MD **2.02 µm more** (0.85 fewer to 4.88 more)
*The risk in the intervention group (and its 95% confidence interval) is based on the assumed risk in the comparison group and the **relative effect** of the intervention (and its 95% CI). **CI:** confidence interval; **MD:** mean difference					
**GRADE Working Group grades of evidence High certainty:** we are very confident that the true effect lies close to that of the estimate of the effect. **Moderate certainty:** we are moderately confident in the effect estimate: the true effect is likely to be close to the estimate of the effect, but there is a possibility that it is substantially different. **Low certainty:** our confidence in the effect estimate is limited: the true effect may be substantially different from the estimate of the effect. **Very low certainty:** we have very little confidence in the effect estimate: the true effect is likely to be substantially different from the estimate of effect.					

a The authors decided to downgrade it as there are many differences in preparation methods, group selection, sample sizes, follow up times and evident presence of publication bias.

b Downgraded because there's even smaller sample size, inconsistent studies, evidence of publication and risk of bias.

### Meta-regression

Results from meta-regression can be found in Table 4.

**Table 4:** Meta-regression.

Predictor	Coefficient	Std. Err.	z	p > *z*	95% CI	
Activator	−10.56124	17.29447	−0.61	0.541	−44.45779	23.3353
Design	39.81193	25.13887	1.58	0.113	−9.459344	89.08321
Spin	−4.740055	18.24044	−0.26	0.795	−40.49065	31.01054
Gender	25.06502	19.76032	1.27	0.205	−13.6645	63.79453
Constant	−52.9052	62.47287	−0.85	0.397	−175.3498	69.53936

R-squared measures the proportion of total variation in the outcome variable that is explained by the predictors in the model. In this case, the value of 0.00% suggests that the predictors included in the model explain very little of the total variation.

The Wald Chi-Square test is used to assess the overall significance of the meta-regression model. In this case, the Chi-Square statistic is 2.68, and the p-value is 0.6134. Since the p-value is greater than the typical significance level of 0.05, the authors fail to reject the null hypothesis, indicating that the overall model is not statistically significant.

## Discussion

Despite high heterogeneity, which limits data pooling by meta-analysis, the findings of this study indicate that PRP therapy can significantly increase hair density compared to placebo. Evidence gathered from the included studies is of low quality, and reports were conducted in various locations, enhancing the external validity of the findings.

One important aspect to highlight is the safety profile of PRP therapy. No intolerable or high-risk adverse effects were reported in the patients included in this research. The most common adverse effect observed was short-duration local pain, which is consistent with previous studies. Roohaninasab M et al., in a review assessing the efficacy, safety, and adverse effects of PRP therapy, also reported high efficacy and low risks associated with the intervention.^51^

These findings are encouraging as they suggest that PRP therapy for AGA is not only effective but also safe. However, it is essential to note that the absence of reported adverse effects in the included studies does not guarantee the absence of potential risks.

### Platelet-rich plasma mechanism

The action of PRP in treating AGA is primarily attributed to the growth factors and cytokines present in the platelet concentrate. These bioactive substances play a crucial role in stimulating anti-inflammatory, angiogenic, and proliferative pathways.^52^ The key factors associated with hair growth include Vascular Endothelial Growth Factor (VEGF), which is upregulated during the anagen phase and plays a role in determining the follicular size and inducing perifollicular angiogenesis; Glial Cell Line-Derived Neurotrophic Factor (GDNF), which suppresses the transition to the catagen phase and exhibits a decline in expression over time during the anagen phase; Insulin-like Growth Factor 1 (IGF-1), which synergistically works with GDNF to promote the proliferation of basal Ki67+ keratinocytes and prolong the anagen phase; and Fibroblast Growth Factor 2 (FGF-2), which promotes dermal papilla proliferation and can be enhanced by adenosine application or in conjunction with nanoparticles.^53^, ^54^, ^55^, ^56^, ^57^, ^58^

Considering the importance of these factors, pre-application regulation of them can be crucial in achieving optimal results. Two studies included in this review examined the role of growth factors in hair regrowth. Siah TW et al. demonstrated a significant association between GDNF and hair density. Additionally, they observed a tendency towards a correlation between VEGF and FGF-2, although this finding may have been limited by the sample size.^40^ Rodrigues BL et al., although not establishing an explicit correlation between Epidermal Growth Factor (EGF), Platelet-Derived Growth Factor (PDGF), VEGF, and aesthetic outcomes, demonstrated an increase in hair density and the presence of anagen phase follicles, which was not observed in the group treated with saline injection alone.^37^

These findings further support the hypothesis of the efficacy of platelet concentrate in promoting hair growth. Moreover, Gentile P et al., although not directly studying growth factors, also observed beneficial effects following PRP application, including epidermal thickening, increased perifollicular vascularization, and an increase in ki67+ keratinocytes, thereby reinforcing the action of PRP.^46^

Qu Q, in his analysis, provided important information on alternative routes after studying mice pelage. He suggests that PRP treatment can activate β-Catenin, PDGF, and AKT signaling pathways, coupled with suppression of p53 levels to induce hair growth.^48^ This provides new insights into future research directions that could lead to a better understanding of hair growth with PRP.

### Preparation methods

The optimal technique for preparing PRP remains a subject of ongoing debate and investigation. In an effort to identify the most effective preparation method, Stevens J et al. conducted a review of the available literature.^59^ Their findings suggested several recommendations, including the use of subdermal bolus injections to minimize discomfort, the incorporation of an activator, monthly treatment intervals, and preparation using the single-spin method. These suggestions aimed to enhance treatment outcomes by optimizing the PRP preparation process.

In contrast with this, our meta-analysis revealed that the use of a pre-application activator suggested slightly lower improvements in hair density when compared to non-active. These findings indicate that pre-activating PRP prior to application may decrease its efficacy in promoting hair regrowth. Despite this, the difference found is very small. Therefore, the authors recommend that future studies explore the duality between pre-activation or not of PRP as a preparation method.

In contrast, the Tawfik AA study employed a different approach, with more frequent injections at weekly intervals using the double-spin method in women with alopecia.^41^ Remarkably, this study demonstrated exceptional results, showing sustained hair growth even after 6 months of treatment. Similarly, Singh S et al. achieved significant outcomes by utilizing the double-spin method while excluding Platelet-Poor Plasma (PPP) and adopting less frequent monthly applications.^43^ These findings suggest that shorter intervals between injections and the utilization of the double-spin method may contribute to more effective hair density improvement. Despite these data, our subgroup meta-analysis of the spin method revealed greater differences with double spin. However, the difference was small between both preparations.

Study design is of utmost importance. It’s interesting to note that our subgroup meta-analysis considering Intra/Inter-subject comparisons presented better results with inter-subject designs. This may be related to PRP spread in the scalp, affecting placebo areas in intra-subject comparisons. Accordingly, research conducted with this design may suggest smaller effects.

Our last subgroup meta-analysis was related to gender, in order to explain the high heterogeneity present. Although it was capable of reducing heterogeneity, the findings were not statistically significant. Additionally, the authors couldn’t ascertain individual-by-individual changes, limiting our analysis capability. More studies should be conducted exclusively on men and women for this issue to be answered.

Considering these findings, it’s apparent that various preparation methods for PRP can yield positive results. Further research is warranted to determine the optimal preparation technique for PRP in hair regrowth treatments. Factors such as injection frequency, activation protocols, and the specific centrifugation method employed may all influence the efficacy of PRP treatment. Future studies should aim to compare different preparation methods and evaluate their impact on hair regrowth outcomes to establish more precise guidelines for clinical practice.

### Current therapy comparison

Globally, the treatment approach for AGA often involves the use of 5-alpha reductase inhibitors, such as finasteride and dutasteride, as well as topical solutions containing minoxidil, an adenosine 5'-triphosphate-sensitive potassium channel opener.^4^, ^60^, ^61^, ^62^ Adil A and Godwin M conducted a systematic review and meta-analysis comparing the effects of these medications against placebo.^63^ The analysis revealed mean differences in hair density compared to placebo as follows: finasteride 1 mg = 18.37 hairs/cm^2^, minoxidil 5% = 14.94 hairs/cm^2^, and 2% minoxidil = 8.11 hairs/cm^2^. Notably, finasteride exhibited high heterogeneity (I^2^ = 91%), while minoxidil demonstrated negligible heterogeneity (I^2^ = 0%).

In the present review, PRP therapy showed a mean difference of 27.55 hairs/cm^2^ compared to placebo, which is higher than the improvement observed with finasteride. However, both PRP and finasteride exhibited high heterogeneity. This improvement is clinically relevant, as PRP provides a notable increase in hair density without the requirement for daily use and the risk of undesired collateral effects. In addition, the PRP mechanism of action differs from other hair treatments, meaning it could be used in a combined therapy, enhancing hair growth results even more. These findings are consistent with the results reported by Gentile P et al., who conducted a systematic review comparing PRP therapy to conventional treatments, as well as with the findings of Singh S et al. and Pakhomova EE, who compared PRP to minoxidil and combined therapies.^43^, ^64^, ^65^ The evidence suggests that PRP therapy is more effective than minoxidil alone, and combination therapy may offer even greater efficacy. This suggests that PRP could be a viable treatment option for AGA.

In summary, PRP therapy has shown promising results in improving hair density in AGA patients. It appears to be a favorable treatment option when compared to finasteride and may provide enhanced efficacy when used in combination with other therapies. These findings support the growing body of evidence highlighting the potential of PRP as a valuable therapeutic approach for managing AGA.

## Limitations

Our meta-analysis integrates the most up-to-date studies accessible within the discipline. By incorporating the most recent research discoveries, the authors have guaranteed that our analysis mirrors the present status of comprehension. This facet holds substantial significance, especially in swiftly developing domains where novel evidence can considerably influence treatment suggestions. The authors have employed sophisticated statistical methods and methodological approaches to augment the exactitude and dependability of our findings. Through cutting-edge meta-analytic and estimate techniques, we’ve mitigated potential biases and heightened the precision of our estimations.

The authors have performed a thorough investigation of different subcategories within the encompassed research. Through the process of categorizing the data according to pertinent variables including age, gender, spin, and activator, the authors have successfully delved into possible disparities in the impact of treatment across diverse populations. This analysis of subgroups yields invaluable perspectives on the diversity of the findings and aids in the identification of particular patient attributes that might impact treatment results.

There’s evident huge heterogeneity in AGA severity between reports. As shown in Table 1, nine papers included high-grade AGA, and five only included patients with less severe situations. Heterogeneity could be attributed both to patient selection and measurement method, in which there is no specification in reports if total hair density or terminal hair density was measured. It’s important to note that some of the reported studies, such as Rodrigues BL et al., had differences in baseline hair density between groups. This certainly influenced in final results, as the PRP group indeed presented a better increase, but couldn’t sufficiently impact to surpass the previous difference between groups. In this sense, Future research may focus on stratifying AGA severity groups for further evidence of PRP efficacy.

Despite the rigorous search process conducted in multiple databases and the inclusion of gray literature, it’s possible that some relevant studies were missed, which could introduce potential bias in the findings.

Another important limitation to consider is the inclusion of intra-subject studies, which may introduce confounding factors. The local-systemic spreading effect of PRP dissemination and the potential impact of regional needling on cellular regeneration pathways could influence the observed outcomes, as suggested by the subgroup meta-analysis. These factors should be taken into account when interpreting the results.^42^, ^66^

Furthermore, it is worth noting that the heterogeneity observed among the included studies, as indicated by the high I² values, may also contribute to limitations in drawing definitive conclusions. Albeit our struggle to group reports to minimize and dive into further explorations on heterogeneity, included studies have small sample sizes, differ in PRP protocols, and present different follow-up times. The variations in final platelet count after centrifugation are another spotlight topic, as most spin methods don’t match in studies, with numerous variations in time and revolutions per minute. AGA severity in studies has deviated even in the same groups, promoting more variability. Our data approach may also have been intoxicated by the previous differences present in groups regarding AGA severity, as reports such as Rodrigues BL et al. presented an evident hair growth in the PRP group, but wasn’t big enough to suppress the control group difference, which didn’t present evident hair growth. These variations in study designs, patient characteristics, and PRP preparation methods certainly influence treatment outcomes and introduce potential sources of bias. Furthermore, there is clear evidence of publication bias as shown by the asymmetry in the funnel plot.

To mitigate these limitations, future research in this area should aim for larger sample sizes, standardized protocols, and longer follow-up periods. Additionally, conducting more studies with a randomized controlled trial design would enhance the quality of evidence and allow for a more comprehensive assessment of the efficacy and safety of PRP therapy for AGA.

Overall, while this systematic review provides valuable insights into the current evidence on PRP therapy for AGA, it is important to consider its limitations and interpret the findings with caution.

## Conclusion

Based on the available evidence from randomized clinical trials, the use of PRP injections for the treatment of AGA has shown effectiveness in increasing hair density. However, it is important to note that the certainty of the evidence is currently at a low level, and further robust studies are needed to strengthen the findings. Finally, this work highlights the lack of high-quality randomized clinical trials. As well as the high heterogeneity and reports of low sample size claims for the need for well-conducted trials on the efficacy of PRP in AGA.

Recommendations for future research include using PRP preparations that exclude platelet-poor plasma, pre-activating PRP before application, and carefully considering confounding factors such as micro-needling or PRP diffusion, when comparing results between individuals. Additionally, future studies should investigate the optimal frequency of PRP treatment intervals, explore the effectiveness of the double-spin method for PRP preparation, and evaluate the potential benefits of combining PRP therapy with other treatment modalities.

Overall, while the current evidence supports the use of PRP injections for AGA, further research is needed to enhance our understanding of its optimal use, improve the certainty of the evidence, and expand our knowledge of potential combination therapies.

## Financial support

None declared.

## Authors’ contributions

Lucas Kieling: The conception and design of the study; Data collection, or analysis and interpretation of data; Statistical analysis; Drafting the article or critically reviewing it for important intellectual content; Obtaining, analyzing and interpreting data; Critical review of the literature; Final approval of the final version of the manuscript.

Ana Terezinha Konzen: The conception and design of the study; Data collection, or analysis and interpretation of data; Statistical analysis; Drafting the article or critically reviewing it for important intellectual content; Obtaining, analyzing and interpreting data; Critical review of the literature; Final approval of the final version of the manuscript.

Rafaela Koehler Zanella: Drafting the article or critically reviewing it for important intellectual content; Obtaining, analyzing and interpreting data; Critical review of the literature; Final approval of the final version of the manuscript.

Denis Souto Valente: Drafting the article or critically reviewing it for important intellectual content; Obtaining, analyzing and interpreting data; Critical review of the literature; Final approval of the final version of the manuscript.

## Conflicts of interest

None declared.
