# Supplementary material for: Is autologous platelet-rich plasma capable of increasing hair density in patients with androgenic alopecia? A systematic review and meta-analysis of randomized clinical trials
Source: An Bras Dermatol. 2024 Jul 15;99(6):847–62. doi: 10.1016/j.abd.2024.01.002 (PMC11551241; doi:10.1016/j.abd.2024.01.002)

**ABD-D-23-00445_ Supplementary material**

**Supplementary material**

| **Complete searches:** |
| --- |
| **MEDLINE (PUBMED)** |
| (((((((Hair regrowth) OR (Hair restoration)) OR (Hair regeneration)) OR (Hair Growth)) AND (((((((((((((((((((((Alopecia) OR (Baldness)) OR (Hair Loss)) OR (Hair Losses)) OR (Loss, Hair)) OR (Losses, Hair)) OR (Alopecia, Male Pattern)) OR (Male Pattern Alopecia)) OR (Baldness, Male Pattern)) OR (Male Pattern Baldness)) OR (Female Pattern Baldness)) OR (Baldness, Female Pattern)) OR (Androgenetic Alopecia)) OR (Pattern Baldness)) OR (Baldness, Pattern)) OR (Androgenic Alopecia)) OR (Alopecia, Androgenic)) OR (Alopecias, Androgenic)) OR (Pseudopelade)) OR (Alopecia Cicatrisata)) OR (Alopecia Cicatrisatas))) AND (((Platelet-Rich Plasma) OR (Plasma, Platelet-Rich)) OR (PRP)))) AND (((randomized controlled trial[pt]) OR (controlled clinical trial[pt]) OR (randomized[tiab] OR randomised[tiab]) OR (placebo[tiab]) OR (drug therapy[sh]) OR (randomly[tiab]) OR (trial[tiab]) OR (groups[tiab])) NOT (animals[mh] NOT humans[mh])) |
|  |
| **EMBASE** |
| 'male type alopecia'/exp AND 'thrombocyte rich plasma'/exp AND 'hair growth'/exp AND 'randomized controlled trial'/exp |
|  |
| **COCHRANE** |
| #1 (Hair regrowth):ti,ab,kw OR (Hair restoration):ti,ab,kw OR (Hair regeneration):ti,ab,kw OR (Hair Growth):ti,ab,kw (Word variations have been searched) 1304 |
| #2 (Alopecia):ti,ab,kw OR (Baldness):ti,ab,kw OR (Hair Loss):ti,ab,kw OR (Hair Losses):ti,ab,kw OR (Losses, Hair):ti,ab,kw 5473 |
| #3 (Alopecia, Male Pattern):ti,ab,kw OR (Male Pattern Alopecia):ti,ab,kw OR (Baldness, Male Pattern):ti,ab,kw OR (Male Pattern Baldness):ti,ab,kw OR (Female Pattern Baldness):ti,ab,kw 223 |
| #4 (Baldness, Female Pattern):ti,ab,kw OR (Androgenetic Alopecia):ti,ab,kw OR (Pattern Baldness):ti,ab,kw OR (Baldness, Pattern):ti,ab,kw OR (Androgenic Alopecia):ti,ab,kw 697 |
| #5 (Alopecia, Androgenic):ti,ab,kw OR (Alopecias, Androgenic):ti,ab,kw OR (Pseudopelade):ti,ab,kw OR (Alopecia Cicatrisata):ti,ab,kw OR (Alopecia Cicatrisatas):ti,ab,kw 209 |
| #6 (Platelet-Rich Plasma):ti,ab,kw OR (Plasma, Platelet-Rich):ti,ab,kw OR (PRP):ti,ab,kw 3914 |
| #7 #2 OR #3 OR #4 OR #5 5473 |
| #8 #1 AND #7 AND #6 84 |
|  |
| **BVS** |
| ((mh:("Randomized Controlled Trials as Topic" OR "Controlled Clinical Trials as Topic" OR "Random Allocation" OR "Double-Blind Method" OR "Single-Blind Method" OR "Placebos" OR "Multicenter Studies as Topic" OR "Cross-Over Studies" OR "Pragmatic Clinical Trials as Topic") OR pt:("Randomized Controlled Trial" OR "Controlled Clinical Trial" OR "Multicenter Studies" OR "Pragmatic Clinical Trial") OR ti:(random* OR aleatori* OR placebo*) OR (ti:("clinical trial" OR "ensayo clinico" OR "ensaio clinico") AND tw:(control* OR random* OR aleatori* OR placebo*)) OR (ti:("cross-Over" OR multicenter OR multicentric*) AND ti:(study OR studies OR estud*)) OR ab:(randomi* OR aleatori* OR placebo*) OR (ab:("clinical trial" OR "ensayo clinico" OR "ensaio clinico") AND tw:(control* OR random* OR aleatori* OR placebo*)) OR (ab:("cross-Over" OR multicenter OR multicentric*) AND ab:(study OR studies OR estud*)) OR (tw:(simple* OR singl* OR duplo* OR doble* OR doubl* OR trebl* OR tripl*) AND tw:(cego OR ciego OR blind OR mask OR dumm*))) AND NOT ((mh:"animals" AND NOT mh:"humans") OR mh:"Retrospective Studies")) AND (((Alopecia) OR (Baldness) OR (Hair Loss) OR (Hair Losses) OR (Loss, Hair) OR (Losses, Hair) OR (Alopecia, Male Pattern) OR (Male Pattern Alopecia) OR (Baldness, Male Pattern) OR (Male Pattern Baldness) OR (Female Pattern Baldness) OR (Baldness, Female Pattern) OR (Androgenetic Alopecia) OR (Pattern Baldness) OR (Baldness, Pattern) OR (Androgenic Alopecia) OR (Alopecia, Androgenic) OR (Alopecias, Androgenic) OR (Pseudopelade) OR (Alopecia Cicatrisata) OR (Alopecia Cicatrisatas)) AND ((Platelet-Rich Plasma) OR (Plasma, Platelet-Rich) OR (PRP) OR (thrombocyte rich plasma)) AND ((Hair regrowth) OR (Hair restoration) OR (Hair regeneration) OR (Hair Growth))) |
|  |
| **WEB OF SCIENCE** |
| (((((((((((((((((((ALL=(Hair Loss)) OR ALL=(Baldness)) OR ALL=(Alopecia)) OR ALL=(Hair Losses)) OR ALL=(Loss, Hair)) OR ALL=(Losses, Hair)) OR ALL=(Alopecia, Male Pattern)) OR ALL=(Male Pattern Alopecia)) OR ALL=(Baldness, Male Pattern)) OR ALL=(Male Pattern Baldness)) OR ALL=(Female Pattern Baldness)) OR ALL=(Baldness, Female Pattern)) OR ALL=(Androgenetic Alopecia)) OR ALL=(Pattern Baldness)) OR ALL=(Baldness, Pattern)) OR ALL=(Androgenic Alopecia)) OR ALL=(Alopecia, Androgenic)) OR ALL=(Alopecias, Androgenic)) OR ALL=(Pseudopelade)) OR ALL=(Alopecia Cicatrisata) |
| (((ALL=(Platelet-Rich Plasma)) OR ALL=(Plasma, Platelet-Rich)) OR ALL=(PRP)) OR ALL=(thrombocyte rich plasma) |
| (((ALL=(Hair regrowth)) OR ALL=(Hair restoration)) OR ALL=(Hair regeneration)) OR ALL=(Hair Growth) |
| (ALL=(Clinical Trial)) OR ALL=(Clinical Trials) |
|  |
| **SCOPUS** |
| ( ( (TITLE-ABS-KEY (alopecia) OR TITLE-ABS-KEY (baldness) OR TITLE-ABS-KEY (hair AND loss) OR TITLE-ABS-KEY (hair AND losses) OR TITLE-ABS-KEY (loss, AND hair) OR TITLE-ABS-KEY (losses, AND hair) OR TITLE-ABS-KEY (alopecia, AND male AND pattern) OR TITLE-ABS-KEY (male AND pattern AND alopecia) OR TITLE-ABS-KEY (baldness, AND male AND pattern) OR TITLE-ABS-KEY (male AND pattern AND baldness) OR TITLE-ABS-KEY (female AND pattern AND baldness) OR TITLE-ABS-KEY (baldness, AND female AND pattern) OR TITLE-ABS-KEY (androgenetic AND alopecia) OR TITLE-ABS-KEY (pattern AND baldness) OR TITLE-ABS-KEY (baldness, AND pattern) OR TITLE-ABS-KEY (androgenic AND alopecia) OR TITLE-ABS-KEY (alopecia, AND androgenic) OR TITLE-ABS-KEY (alopecias, AND androgenic) OR TITLE-ABS-KEY (pseudopelade) OR TITLE-ABS-KEY (alopecia AND cicatrisata) OR TITLE-ABS-KEY (alopecia AND cicatrisatas) ) ) AND ( (TITLE-ABS-KEY (platelet-rich AND plasma ) OR TITLE-ABS-KEY (plasma, AND platelet-rich ) OR TITLE-ABS-KEY (prp) OR TITLE-ABS-KEY (thrombocyte AND rich AND plasma) ) ) AND ( (TITLE-ABS-KEY (hair AND regrowth) OR TITLE-ABS-KEY (hair AND restoration) OR TITLE-ABS-KEY (hair AND regeneration) OR TITLE-ABS-KEY (hair AND growth) ) ) ) AND (TITLE-ABS-KEY (clinical AND trial) ) |
|  |
| **medRxiv** |
| “Alopecia” AND “Platelet-Rich Plasma” AND “randomized clinical trial” |
|  |
| Articles excluded from the systematic review according to the exclusion criterion. |

**Subgroup meta-analysis on study design.**

| **Author, year** | **Rationale / Criterion for exclusion** |
| --- | --- |
| Sasaki, 2021 | Sample <10 patients |
| Puig, 2016 | Absence of hair density |
| Hausauer, 2018 | No placebo |
| Bayat, 2019 | No placebo |
| Kapoor, 2020 | No placebo |
| Ince, 2018 | No placebo |
| Takikawa, 2011 | Not randomized |
| Mapar, 2016 | Absence of hair density |
| NCT03474718 | Protocol |
| Kumar, 2020 | Abstract |
| Abaroa, 2016 | Abstract |
| Tan, 2019 | Abstract |

**Subgroup meta-analysis on study design.**


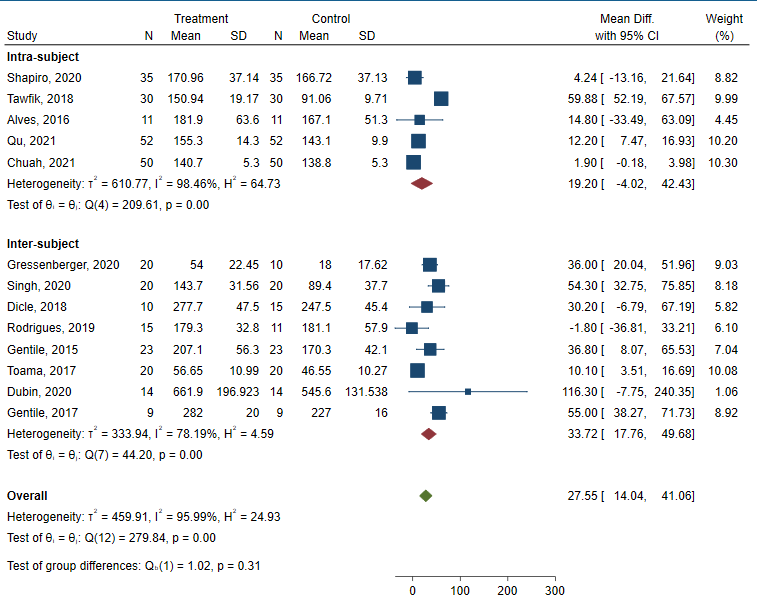


**Subgroup meta-analysis on gender.**


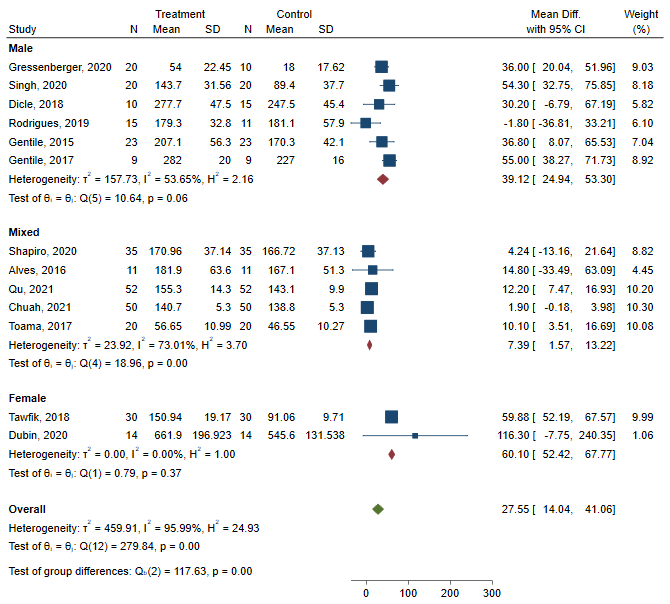


**Subgroup meta-analysis on spin.**


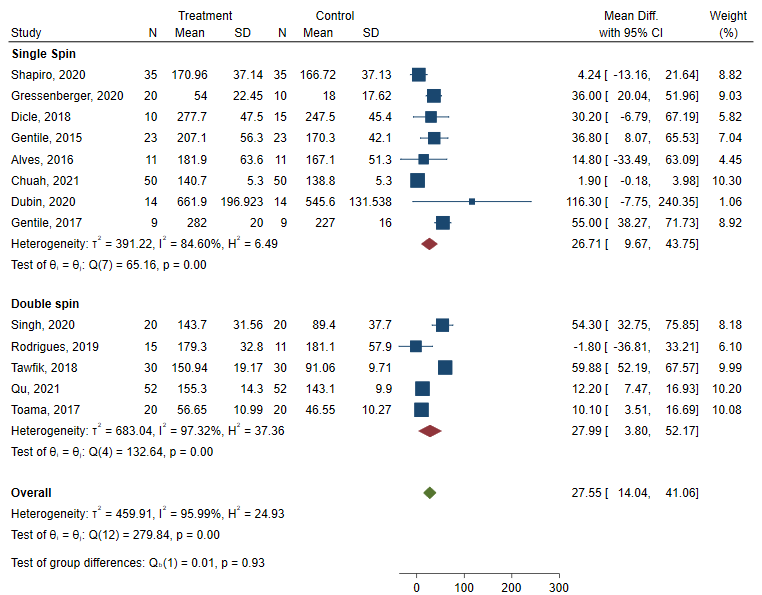


**Subgroup meta-analysis on activator.**

**
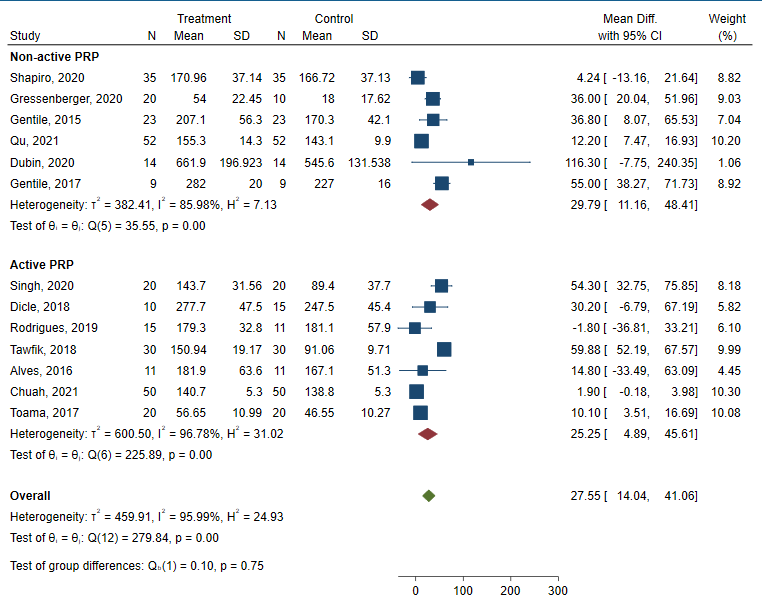
**

**Subgroup meta-analysis on risk of bias.**

**
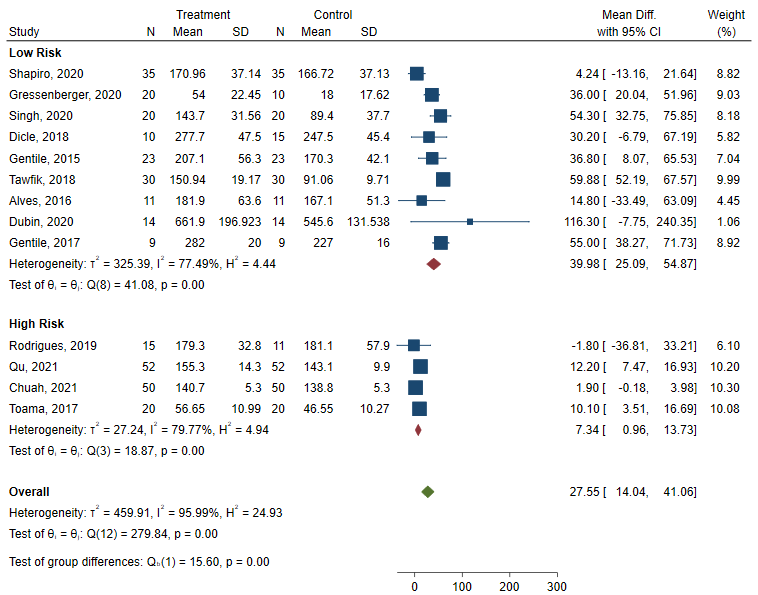
**

**Funnel plot Gender.**


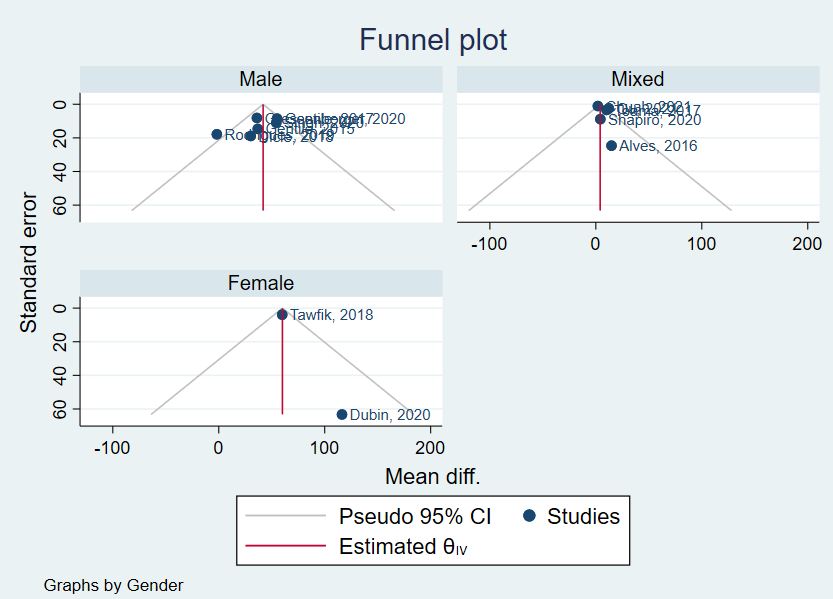


**Funnel Plot Activator.**


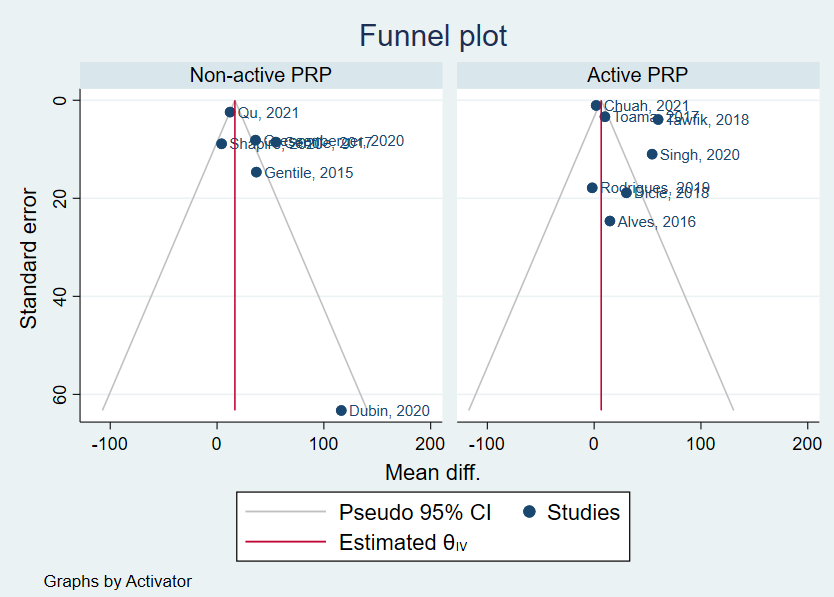


**Funnel plot design.**


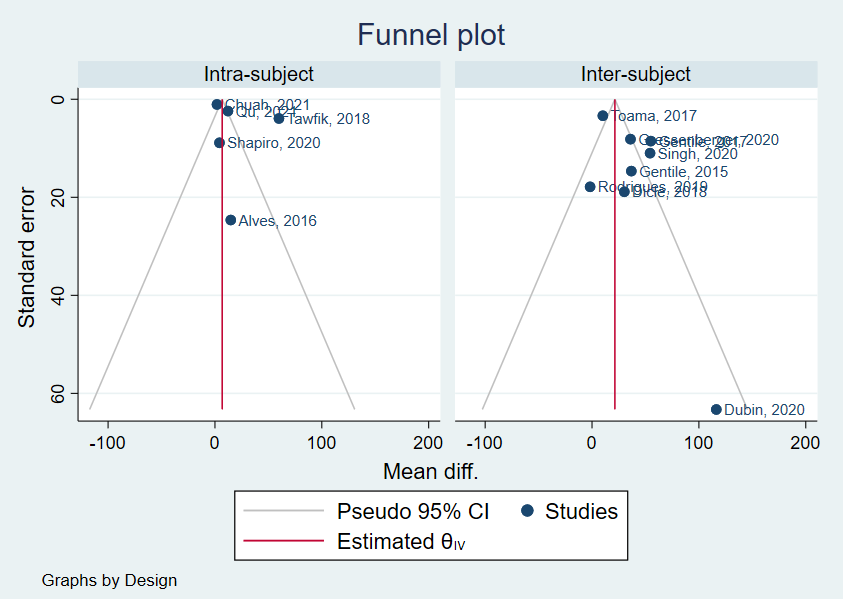


**Funnel plot spin.**


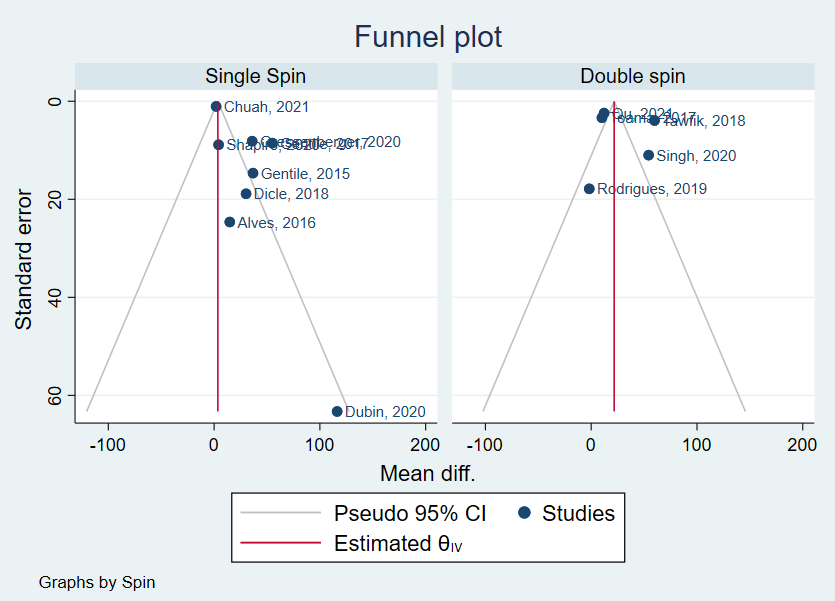

Supplement: Supplementary file 1 [file mmc1.docx]
